# Whole Wheat Flour

**Published:** 1875-12

**Authors:** 


### WHOLE WHEAT FLOUR.

No whole wheat flour which we have ever examined, equals the Cold-air Attrition Flour, in the perfection of the pulverizing process. It is very clean, not dark-colored, because made from very choice, plump wheat; and the bread and cake made from it is sweet and delicious. It is not like "Graham," to be used *with* other bread foods, but is to be used *instead* of all other breads. It is just the thing for mothers to bring up children upon. It is not merely a food for dyspeptics, but a food for well folks to keep well upon.

